# Annual level changes of serum neuronal and glial biomarkers in a German professional football club

**DOI:** 10.1007/s00415-025-13176-z

**Published:** 2025-06-13

**Authors:** Robert Marshall, Samir Abu-Rumeileh, Lisa Habeck, Petra Steinacker, Matteo Foschi, Kai Wohlfahrt, René Schwesig, Helge Riepenhof, Jan-Niklas Droste, Lorenzo Barba, Markus Otto

**Affiliations:** 1 RasenBallsport Leipzig GmbH, Cottaweg 3, 04177 Leipzig, Germany; 2 Department of Orthopaedic and Trauma Surgery, Martin-Luther-University Halle-Wittenberg, Ernst-Grube-Strasse 40, 06108 Halle (Saale), Germany; 3 Department of Neurology, Martin-Luther-University of Halle-Wittenberg, Halle (Saale), Germany; 4 Department of Biotechnological and Applied Clinical Sciences, University of L’Aquila, L’Aquila, Italy; 5 Department of Neuroscience, Neurology Unit, S.Maria Delle Croci Hospital of Ravenna, AUSL Romagna, Ravenna, Italy; 6 Department of Neurology, BG Hospital Bergmannstrost, Halle (Saale), Germany; 7 Center for Rehabilitation and Sports Medicine, BG Klinikum Hamburg, 21033 Hamburg, Germany; 8 Red Bull Athlete Performance Center Thalgau, 5303 Salzburg, Austria; 9 RedBull Pro Cycling GmbH & Co KG, Innstraße 1, 6342 Niederndorf, Austria

**Keywords:** Football, neurofilament light chain, glial fibrillary acidic protein, head trauma, concussion, sport

## Abstract

**Background:**

Professional football players (PFP) experience repeated mild traumatic brain injuries (TBI) and have an increased long-term dementia risk. We aimed to assess annual level changes of blood neuronal (neurofilament light chain, NfL) and astroglial (glial fibrillary acidic protein, GFAP) biomarkers in PFPs over 2 years.

**Methods:**

We measured with commercial immunoassays NfL and GFAP concentrations *n* = 129 serum samples obtained from *n* = 43 male PFPs playing for a German professional football team. Samples were collected at five time points over 2 years and before/after an index match. Associations between blood markers and potential sources of neuronal damage, such as intense physical activity, injuries, and headers, were tested.

**Results:**

Serum NfL and GFAP concentrations in PFPs were significantly different at repeated measurements (*p* < 0.001) but were not associated with metrics of physical activity, total time of physical activity, total number of headers, and headers-per-match. After injuries with mild TBI, serum NfL and GFAP increased and returned to normal levels within few days. Before and after an index match, serum levels of NfL and GFAP were not significantly different, nor they were significantly associated with physical activity and headers.

**Discussion:**

Serum NfL and GFAP may be used to monitor PFP over time. Repeated headers and intense physical activity in PFPs seem to be safe on a neurochemical level.

**Supplementary Information:**

The online version contains supplementary material available at 10.1007/s00415-025-13176-z.

## Introduction

Sport-related concussion (SRC) represents a mild subtype of traumatic brain injury (TBI) occurring in several contact sports, including European football (soccer). Traumatic axonal injury, petechial haemorrhages, microglial clusters, and deposition of aggregation-prone proteins such as amyloid-β and tau are common pathological findings after chronic low-intensity brain damage [1]. The neurochemical correlates of SRC have been studied in team sports involving physical contact, such as American [2, 3] and Australian football [4], but remain poorly explored in European football.

To date, it is still unclear whether chronic subclinical mild TBI may pose a long-term risk increase for neurodegenerative diseases [5], and the identification of individuals at risk despite the absence of clinical symptoms after mild TBI remains challenging for health professionals caring for athletes [6, 7]. Given the worldwide diffusion of interest in European football and given recent evidence of increased incidence of dementia in professional European football players (PFPs) [8], safety concerns about the neurological risk of this sport (with and without head impacts) should be better addressed.

**Fig. 1:**
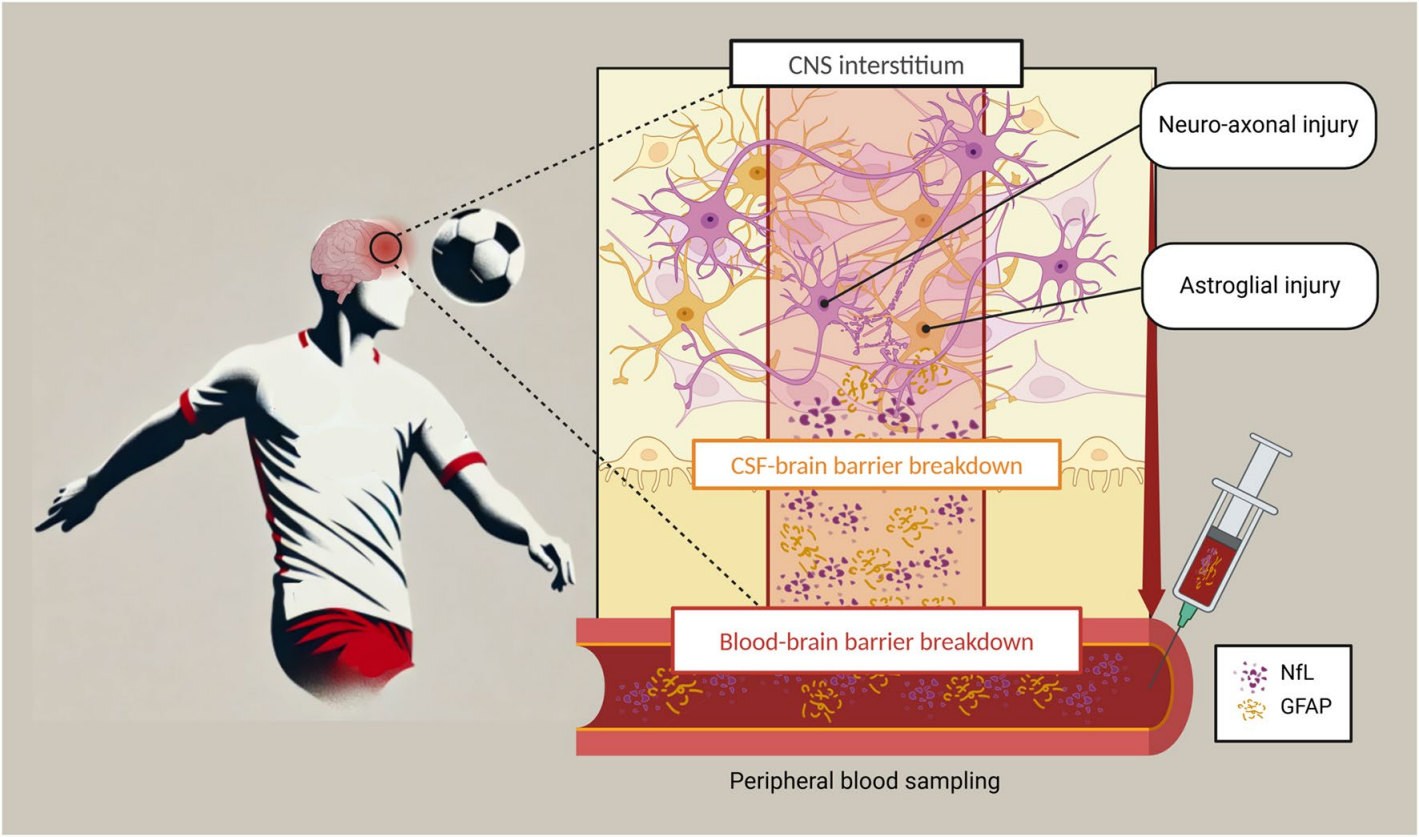
Biomarker release after head trauma in professional football players. After head impacts (both headers and traumatic head injury), neuronal damage and glial activation can occur, leading to protein release from CNS interstitium to peripheral blood through the blood–brain barrier. Blood-based neuronal and glial biomarkers can be used to and monitor signs of structural brain injury to monitor football players on an individual level

Here, fluid biomarkers may help clinicians to recognise signs of structural brain damage after TBI (Fig. 1). Blood-based biomarkers reflecting neuronal and astroglial injury, such as neurofilament light chain (NfL) and glial fibrillary acidic protein (GFAP), have been exploited in healthy subjects and a variety of neurological disorders including TBI [9–11]. NfL levels in serum (sNfL) of PFPs were shown to be of comparable range to those of healthy age-matched controls [12] and were not significantly associated with the kinetics of head impacts [13, 14]. However, other authors have found opposite results, namely higher levels in football players compared to control subjects [15, 16]. On the other hand, GFAP has been investigated only in cerebrospinal fluid (CSF) samples of amateur football players and found to be not associated with headings [17], whereas data on blood GFAP in PFPs are lacking. Moreover, it is unknown whether NfL and GFAP concentrations in blood of PFPs may show seasonal changes in relation to the intensity of physical activity and/or head impacts.

To evaluate these issues, we investigated PFPs at a clinical and neurochemical level in a German professional football club over a time period of approximately 2 years. Since most data on blood biomarker research in PFPs come from cross-sectional studies, we assessed the longitudinal trajectories of sNfL and serum GFAP (sGFAP) by repeated blood sampling to detect seasonal changes in biomarker levels. Second, we collected several parameters of global physical activity and potential sources of brain injury, such as repeated headers, to test their associations with serum biomarkers. Third, we investigated whether changes in biomarker levels were detectable in short-term periods before and after an index football match and whether such changes were associated with physical activity and/or headers.

## Methods

### Study protocol

**Fig. 2:**
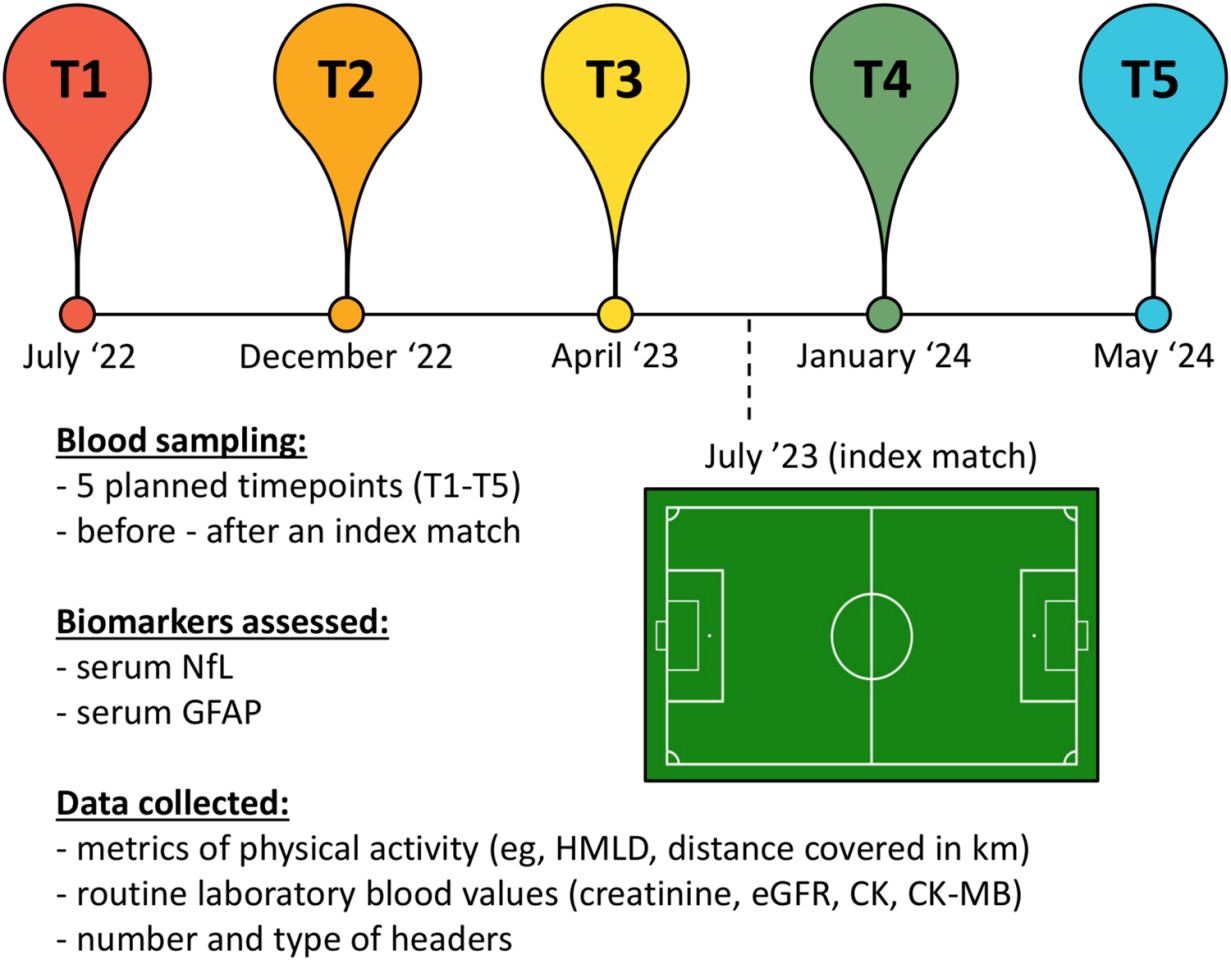
Study protocol. Blood samples were collected at five planned time points (T1-T5) across two regular seasons and before/after an index match between the two seasons. From each blood sample, we measured sNfL, sGFAP, and routine laboratory parameters (creatinine, eGFR, CK, and CK-MB). Along the monitoring period, we collected anthropometric data and metrics of physical activity (e.g., minutes of play, HMLD, etc.), as well as the number and type of headers. *CK* creatine kinase, *eGFR* estimated glomerular filtration rate, *HMLD* high metabolic load distance, *sGFAP* serum glial fibrillary acidic protein, *sNfL* serum neurofilament light chain

In this study, we collected 129 serum samples obtained from 43 male PFPs (cohort details in Table 1). PFPs were enrolled from a German professional football team and competed in the top national league (Bundesliga), in the German national cup tournament (DFB-Pokal) as well as in the professional Union of European Football Associations (UEFA) Champions League and the Fédération Internationale de Football Association (FIFA) World Cup. Biosamples of PFPs were collected at five different time points during two seasons (2022/2023 and 2023/2024) (Fig. 2). Specifically, we first collected blood samples in the 2022 pre-season (July 2022, T1), in the middle (December 2022, T2), and at the end (April 2023, T3) of the 2022/2023 season. For the following period, we collected samples in the middle (January 2024, T4) and at the end (May 2024, T5) of the 2023/2024 season. Further, we performed two additional blood sample collections before and after an index match during the 2023 pre-season retreat (July 2023) (study protocol described in Fig. 2). Complete data were available for *n* = 25 PFPs for the 2022/2023 season, *n* = 23 PFPs for the 2023/2024 season, and *n* = 10 PFPs for the index match.

**Table 1:** Demographic, antropometric and biochemical data of the study population consisting of 43 male professional football players (PFPs)

	Variable	PFP (*n* = 43)
Demographics	Age [mean (± sd)]	23.2 (± 4.8)
Position [*n* (%)]	goalkeeper (GK)	6 (14.0)
	defender (DEF)	15 (34.9)
	midfielder (MID)	14 (32.6)
	striker (ST)	8 (18.5)
Anthropometry	Height [cm]	183 (179–189)
	Weight [kg]	80.6 (75.8–85.7)
	BMI [kg/m^2^]	23.6 (22.9–24.8)
Blood analysis	eGFR at baseline [ml/min/1.73 m^2^]	88 (85–97)
sNfL [pg/ml]	T1	9.8 (7.0–11.0)
	T2	10.0 (8.1–13.0)
	T3	10.6 (7.3–14.0)
	T4	3.6 (2.2–6.8)
	T5	8.2 (4.9–10.85)
sGFAP [pg/ml]	T1	57.5 (52.1–72.7)
	T2	75.3 (66.1–93.0)
	T3	67.3 (54.4–76.1)
	T4	75.1 (65.6–88.6)
	T5	75.4 (63.3–89.1)

Continuous variables are reported as median (interquartile range) except for age (reported as mean and standard deviation)

*BMI* body mass index, *eGFR* estimated glomerular filtration rate, *sGFAP* serum glial fibrillary acidic protein, s*NfL* serum neurofilament light chain, *PFP* professional football players

### Clinical and biochemical data

The first blood sampling, as well as the collection of demographic and anthropometric data of the participating PFPs, took place during the regular medical check-up at the beginning of the pre-season (T1) (Table 1). For each time point of biomarker quantification, we also had data on other blood parameters, namely, serum creatinine level, creatine kinase (CK), and creatine-kinase muscle/brain (CK-MB). We calculated the estimated glomerular filtration rate (eGFR) according to well-established formulas based on serum creatinine: eGFR = 141 × min(S_cr_/κ, 1)^α^ × max(S_cr_/κ, 1)^−1.209^ × 0.993^Age^ where S_cr_ is standardised serum creatinine in mg/dl, κ and α are 0.9 and − 0.302, respectively (all PFPs were male), min indicates the minimum of S_cr_/κ or 1, and max indicates the maximum of S_cr_/κ or 1 [18]. Data on eGFR were expressed as ml/min/1.73 m^2^. Regarding physical metrics, we collected data on overall training and match time (in minutes and days), total running distance (in km), measures of high-speed running (high metabolic load distance, HMLD, defined as the distance covered with a metabolic power of at least 25.5 W/kg, i.e., with speed > 5.5 m/s and acceleration/deceleration > 2 m/s^2^ for at least 1 s), and the number of headers during official matches as a proxy of athletes’ playing style and overall header impact. For the index match, we also collected data on the total activity time, number, and type of headers (short, middle, and long range) (Fig. 2).

### Blood sample collection and biomarker analysis

Serum samples were collected by the medical staff of RasenBall Leipzig (Leipzig, Germany) and processed according to standardised procedures. Samples were aliquoted (500 μL) in sterile polypropylene and stored at − 80 °C until analysis. For biomarker quantification, we purchased commercially available immunoassays run on the ELLA microfluidic system platform for sNfL (BioTechne, Minneapolis, USA) and on an HD-X Simoa platform for sGFAP (Quanterix Inc., Lexington, USA). Internal controls were measured on all plates to quantify the intra- and inter-assay coefficients of variability (CVs) which were < 15% and < 20% for all measurements, respectively.

### Statistical analysis

Statistical analyses were performed using R studio v.4.2.2 (R Foundation, Vienna, Austria) and GraphPad v.8 (GraphPad Software, La Jolla, USA). Chi-squared and Mann–Whitney *U* tests were used to compare categorical and continuous variables among two groups, respectively. The Kruskal–Wallis test was used to compare more than two groups. Correlations were analysed with the Spearman’s rho coefficient and interpreted as negligible (< 0.1), weak (0.1–0.4), moderate (0.4–0.7), strong (0.7–0.9), and very strong (> 0.9) [19]. A correlation of *r* > 0.7 (explained variance > 50%) was considered relevant and marked in bold. Regarding the sample size of *n* = 43, the critical value for the product moment correlation based on a two-sided t test and *a* = 5% is *r* = 0.300 [20]. We built mixed-effects models with the Geisser–Greenhouse correction and Tukey’s multiple comparison tests to detect differences in biomarker concentrations at different time points. We tested the biomarker levels both as absolute concentrations and as annual [(level in T3/level in T1 × 100) – 100] or biannual [(level in T5/first available level × 100) – 100] level change (in %). The Wilcoxon matched-pairs signed-rank test was used for pairwise comparison of biomarker concentrations before and after the index match. Statistical significance was set at *p* values < 0.05.

### Study protocol approvals

This study was conducted in accordance with the Helsinki Declaration and its recent modifications. Written informed consent was obtained from all participants and the Ethics Committee of Halle University approved the study protocol (registry number: 2021–101).

### Data availability

Anonymized data will be shared with qualified investigators on reasonable request to the corresponding author.

## Results

### Study cohort

**Fig. 3:**
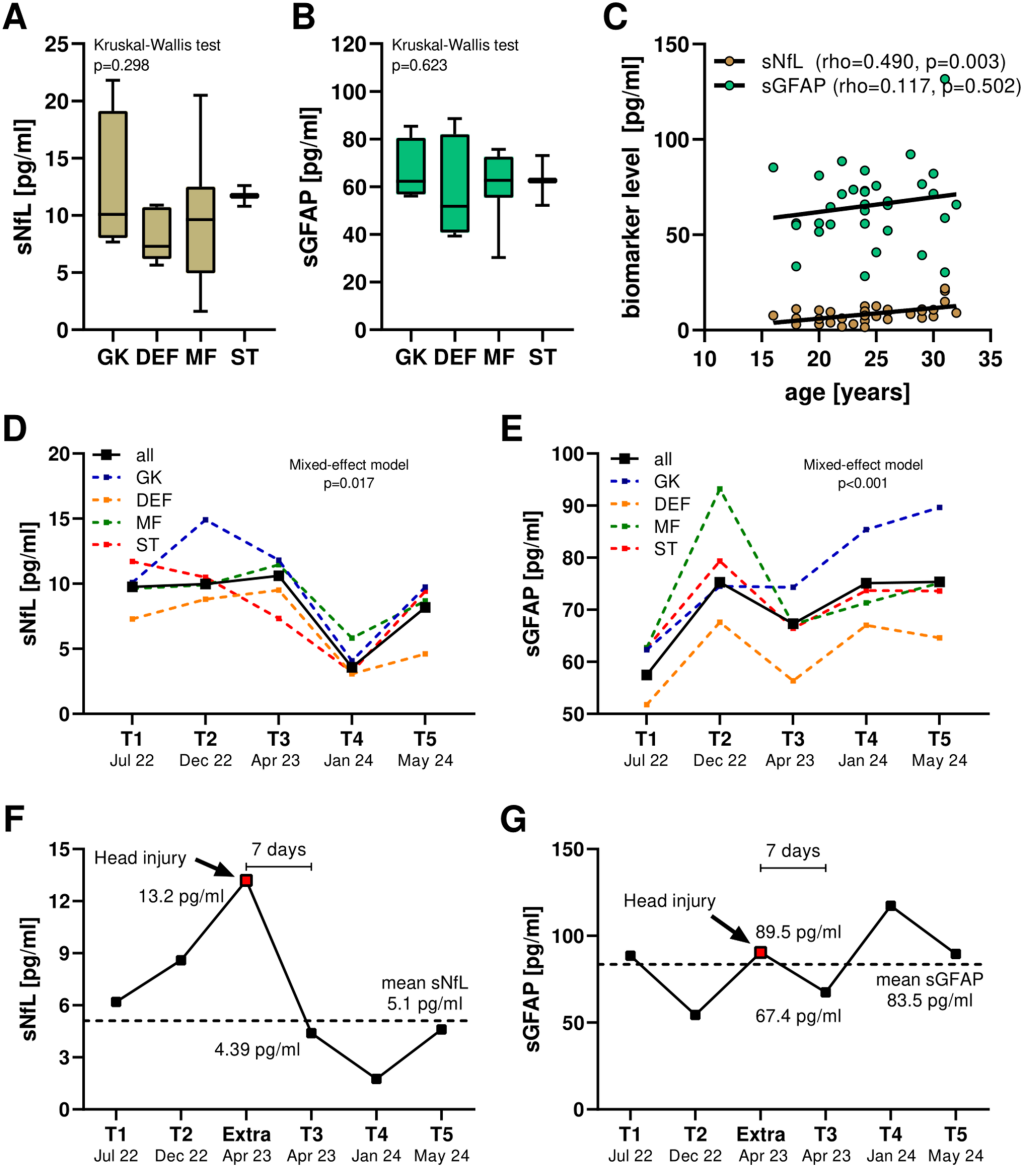
Serum NfL and GFAP in professional football players (PFPs). **A**, **B** Serum markers in PFPs playing in different positions. **C** Correlations between serum markers and age. **D**, **E** Annual level changes of sNfL and sGFAP in PFPs across two regular seasons. **F**, **G** Case report of a 24 years old male PFP who had a traumatic head injury during an official match and received an additional blood sampling 24 h after injury. This occurred 7 days before the planned collective blood sampling at T3. The PFP showed within few days full recovery without clinical sequelae and, at T3, reduced biomarker values. *sGFAP* serum glial fibrillary acidic protein; *sNfL* serum neurofilament light chain

Our cohort included 43 male PFPs [mean age: 23.2 (± 4.8) years] (Table 1). Amongst PFPs, we included 6 goalkeepers (GK, 14.0%), 15 defenders (DEF, 34.9%), 14 midfielders (MID, 32.6%), and 8 strikers (ST, 18.5%). None of the PFPs had a previous history of clinically relevant medical conditions, and the median eGFR values at baseline were 88 ml/min/1.73 m^2^ (interquartile range, IQR: 85–97 ml/min/1.73 m^2^). We found no significant differences in sNfL (*p* = 0.298) and sGFAP (*p* = 0.623) levels between PFPs playing in different positions (Fig. 3A). In PFPs, an older age was significantly correlated with higher sNfL concentrations (Spearman rho = 0.490, *p* = 0.003) but not with sGFAP concentrations (rho = 0.117, *p* = 0.502) (Fig. 3B). In addition, neither sNfL nor sGFAP were significantly correlated with height, weight, or BMI at baseline.

### Longitudinal changes of serum biomarker levels

Repeated measurements of serum biomarkers in PFPs revealed significant differences in sNfL and sGFAP concentrations over the 2-year observation period (mixed-model effect *p* = 0.017 and *p* < 0.001, respectively), with similar results for players in different roles (Fig. 3C). During the first season, median sNfL level remained stable from T1 (July 2022) to T3 (April 2023), whereas it was significantly lower in T4 (January 2024, Tukey’s *p* = 0.0497 vs. T2 and *p* = 0.035 vs. T3). Instead, sGFAP increased significantly from T1 (July 2022) to T2 (December 2022, *p* < 0.001) and from T3 (April 2023) to T5 (May 2025, *p* < 0.001) (Fig. 3C). In PFPs, the correlations of sNfL and sGFAP with each other and with eGFR, CK, or CK-MB (each measured the same day as NfL and GFAP) were not significant at any time point. Similarly, we did not observe relevant correlations between the annual level change [(concentration in T3/concentrations in T1) × 100–100] (in %) of sNfL and sGFAP with each other or with other blood parameters.

### Case reports of head injuries

During seasonal monitoring, we collected a blood sample from a 22-year-old male PFP 24 h after a head injury occurred during an official league match. sNfL and sGFAP concentrations were available for all time points over 2 years with mean concentrations of sNfL 5.1 pg/ml and sGFAP 83.5 pg/ml. Serum samples were obtained 24 h after TBI and measured concentrations were 13.2 pg/ml for sNfL and 89.5 pg/ml for sGFAP (Fig. 3D). Within a few days, the PFP fully recovered and returned to training without any physical, neurological, or psychological symptoms. The injury occurred 7 days before the collective blood sampling at T3 (Fig. 3D). At T3, the concentrations of sNfL (4.39 pg/ml) and sGFAP (67.4 pg/ml) were lower than those measured at the time of TBI. We also collected serum samples also from another PFP who suffered head injury during the monitoring period but for whom serum samples were not available at all time points (Supplementary Figure S2).

### Associations between serum biomarkers and physical activity

We found no relevant correlations between absolute sNfL or sGFAP absolute concentrations at the end of the first season (i.e., T3) and metrics of physical activity, namely total distance covered per season (in km), HMLD units, number of matches or minutes played, as well as the total time of physical activity recorded (in minutes). Similar results were observed when analysing data from the second season, when merging data from the two seasons and when considering the annual level change (in %) of sNfL and sGFAP concentrations.

### Associations between serum biomarkers and headers

During the first season, end-of-season sNfL and sGFAP concentrations were not relevantly correlated with the number of headers (i.e., total number during the season, headers-per-match, and headers per 90 min played) (Supplementary Table S1). Correlations were also not relevant also when considering the annual change in biomarker levels. We did not observe relevant correlations between biomarker concentrations and number of headers neither in the second season nor when considering the two seasons together (full data in Supplementary Table S1).

### Serum biomarkers before and after an index match

**Fig. 4:**
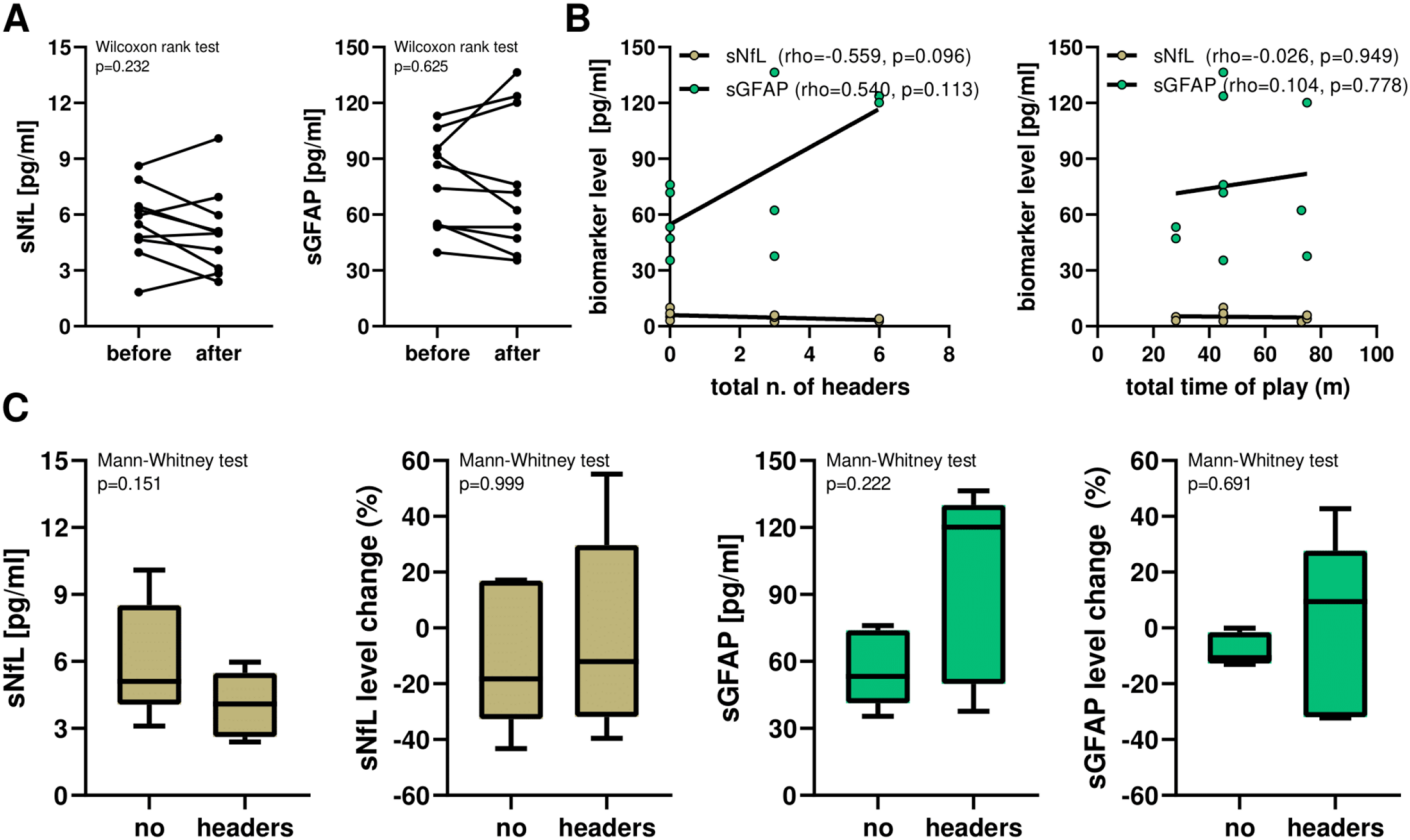
Biomarker levels before and after an index match. **A** sNfL and sGFAP in PFPs before and after the index match. **B** Correlations between biomarkers and total time of play in minutes. **C** Serum biomarker levels and level change (in %) and in PFPs with vs. without headers. *PFP* professional football players, *sGFAP* serum glial fibrillary acidic protein, *sNfL* serum neurofilament light chain

We collected data from *n* = 10 PFPs before and after an index match to observe whether changes in biomarker levels could be detected in a short period of time. We found no significant differences in sNfL and sGFAP concentrations in serum samples collected before and after the match (Wilcoxon matched-pairs signed-rank test *p* = 0.232 for sNfL and *p* = 0.625 for sGFAP) (Fig. 4A). Furthermore, we did not find any relevant correlations between the biomarker concentrations measured after the match and the total playing time (in minutes) (Fig. 4B). Regarding the headers, we did not observe significant associations between the absolute concentrations or changes of sNfL and sGFAP levels before and after the match and the number of headers, i.e., no significant differences between PFPs with and without headers and no significant correlations with the number of headers (Fig. 4C). sNfL (rho = 0.867) and sGFAP (rho = 0.879) concentrations measured before the match were well correlated with those measured after the match. Instead, sNfL and sGFAP levels were not significantly correlated with each other neither before (rho = − 0.624, *p* = 0.060) or after the match (rho = − 0.406, *p* = 0.247).

## Discussion

In this study, we investigated the longitudinal changes in biomarker blood levels of PFPs over two full seasons in relation to parameters of physical activity and head impact. We found significant fluctuations in biomarker levels during different periods of each season and a distinct temporal profile of sNfL and sGFAP, which could be used for individual-level monitoring of PFPs. Furthermore, repeated head impacts in the form of headers during two seasons were not associated with significant changes in biomarker levels that would reflect neuronal and glial injury compared to previous data on TBI patients [21]. In our cohort, the physical effort and the number of headers recorded correspond to the published average exposure of professional European footballers [22, 23]. Our results on heading activity by playing position (in descending order: defender, midfielder, striker, and goalkeeper) are consistent with the previous research [22, 24]. Thus, the population selected for this study can be considered representative of professional European football.

These findings add new insights to understand SRC in professional football on a neurochemical basis. We were able to confirm the previous results on NfL from cross-sectional and longitudinal studies [12–14] and additionally found, in addition, that sNfL concentration does not correlate relevantly with the increase of high-intensity physical activity assessed longitudinally as well as with repeated head impacts. Controversially, other authors reported increased blood NfL levels after headers in football [15, 16]. However, these studies tested head impacts in pre-defined experimental conditions and were therefore less likely to reflect real-world settings in professional football. Interestingly, data on PFPs differ from those obtained in other sports with repeated head impacts, such as American football, where sNfL levels appear to increase longitudinally from the beginning to the end of each season associated with head impacts [25]. The discrepancies in serum biomarker levels between different sports (e.g., a longitudinal increase in American football over a season versus no increase in European football) therefore raise concerns about the safety level of repeated head impacts in professional athletes of the respective sports. The kinematics of head impacts and their voluntary anticipation could contribute significantly to subclinical, but laboratory chemically relevant, brain tissue damage, which varies greatly depending on the sport [26–30]. These findings suggests that different types of sport may need to be assessed differently due to their individual risk profiles.

On another level, we provided the first detailed data on sGFAP and its seasonal changes in PFPs playing at the highest level worldwide (including the UEFA Champions League and FIFA World Cup). Previous data showed no abnormal changes in CSF GFAP levels measured 7–10 days after head impacts [17]. However, as mentioned above, the experimental conditions were pre-determined and no PFPs were tested on an observational basis. Moreover, the temporal course of blood GFAP concentrations after TBI appears to be relatively rapid, i.e., with increase/decrease of marker concentrations within hours or a few days from the trauma [21]. In our cohort, the lack of association of sGFAP levels with an increasing number of head impacts (both during the season and after an index match) suggests that most contacts in professional football do not cause relevant glial activation/injury that can be detected biochemically in peripheral blood. Furthermore, it is noteworthy that sGFAP levels did not correlate relevantly with sNfL concentrations at any time of biomarker quantification. This observation suggests that serum levels of such biomarkers in healthy highly active individuals may reflect physiological mechanisms (e.g., physiological protein turnover) that are different from those in neurological diseases (e.g. neuronal damage and astroglial activation) [10, 11]. Hence, our results suggest that high-intensity physical activity and repeated headers in European professional football are not associated with a significant increase in sNfL and sGFAP levels. Although no population-level effects can be detected in PFPs, blood biomarkers of neuronal and glial injury could be used to monitor PFPs periodically at the individual level to better assess the severity of SRC after head impacts and potentially monitor the recovery process [9]. Therefore, the previous evidence of increased risk of neurodegenerative disorders in former PFPs [8, 31] cannot be explained solely by the higher burden of neuronal/glial damage, as reflected by blood markers, during the active years at a professional level.

As a limitation of our study, we acknowledge the lack of data on the dynamics of head impacts during the observed seasons. Although of interest, such data under controlled experimental conditions have previously shown that head-impact kinetics do not significantly influence sNfL concentrations [13]. Second, none of the PFPs in our cohort were severely injured or had clinically relevant neurological or psychiatric symptoms during the follow-up period. However, we did not perform a thorough neuropsychological assessment for disorders suggestive of post-concussive syndrome, such as fatigue, anxiety, irritability, impulsivity, demotivation, and others. Therefore, future studies should better evaluate the possible predictive value of blood biomarkers for SRC sequelae and other mid- and long-term clinical outcomes (return-to-play time, future development of neurological disorders). Third, we did not examine associations between serum biomarker levels and neuroimaging, which can reveal slight pathological findings (especially on magnetic resonance imaging, MRI), such as traumatic and/or diffuse axonal injury, in about one-quarter of subjects after mild TBI [32]. Given the subtle biomarker-level changes in healthy young adults without major trauma and the lack of non-PFP control subjects in our cohort, it should be further evaluated whether large normalised values on reference control populations could be used for identifying pathological biomarker-level changes at individual level [33, 34]. Fourth, we assessed two well-established biomarkers (NfL and GFAP), but other pathophysiological aspects, such as synaptic dysfunction, neuroinflammation, and early degenerative processes, are still understudied in TBI [35]. Fifth, it should be better evaluated on a larger scale whether these results on mild TBI could be replicated longitudinally in other professional football teams and in amateur football players, in other sports, as well as in diseased individuals [35].

In conclusion, sNfL and sGFAP showed seasonal variations in PFPs over two years and did not correlate with increased physical activity or a higher number of headers. Furthermore, no significant differences between sNfL and sGFAP were found before and after an index match. Head impact in professional football does not appear to increase serum marker levels reflecting brain injury on a 2-year basis. Future studies should better evaluate longer follow-up periods for long-term clinical outcomes.

## Supplementary Information

Below is the link to the electronic supplementary material.

Supplementary file1 (DOCX 13 KB)

Supplementary file1 (TIFF 122 KB)
