# Supplementary material for: Annual level changes of serum neuronal and glial biomarkers in a German professional football club
Source: J Neurol. 2025 Jun 13;272(7):461. doi: 10.1007/s00415-025-13176-z (PMC12165892; doi:10.1007/s00415-025-13176-z)
Supplement: Supplementary file 1 — Supplementary file1 (DOCX 13 KB) [file 415_2025_13176_MOESM1_ESM.docx]

**Supp. Figure S1. Serum biomarker levels in a professional football player after head injury.**

**Supplementary Table S1.** Correlations between serum biomarker levels and number of headers across two seasons.

| **2022/2023 season** | **Total headers** | **N. of headers per match** | **N. headers per 90 minutes played** |
| --- | --- | --- | --- |
| end-season NfL (T3) | rho = 0.075 | rho = -0.024 | rho = -0.157 |
| end-season GFAP (T3) | rho = -0.118 | rho = -0.140 | rho = -0.161 |
| annual NfL level change (%) | rho = 0.090 | rho = 0.098 | rho = -0.004 |
| annual GFAP level change (%) | rho = -0.004 | rho = -0.008 | rho = 0.124 |
| **2023/2024 season** |  |  |  |
| end-season NfL (T5) | rho = -0.401 | rho = -0.412 | rho = -0.377 |
| end-season GFAP (T5) | rho = 0.109 | rho = 0.077 | rho = -0.241 |
| **both seasons** |  |  |  |
| end-season NfL (T5) | rho = -0.189 | rho = -0.187 | rho = -0.062 |
| end-season GFAP (T5) | rho = -0.285 | rho = -0.270 | rho = -0.295 |
| biannual NfL level change (%) | rho = -0.057 | rho = -0.071 | rho = -0.100 |
| biannual GFAP level change (%) | rho = 0.244 | rho = 0.234 | rho = 0.070 |

Correlations were not significant at predefined threshold of p<0.05.
