# Supplementary figures and images for: Annual level changes of serum neuronal and glial biomarkers in a German professional football club
Source: J Neurol. 2025 Jun 13;272(7):461. doi: 10.1007/s00415-025-13176-z (PMC12165892; doi:10.1007/s00415-025-13176-z)

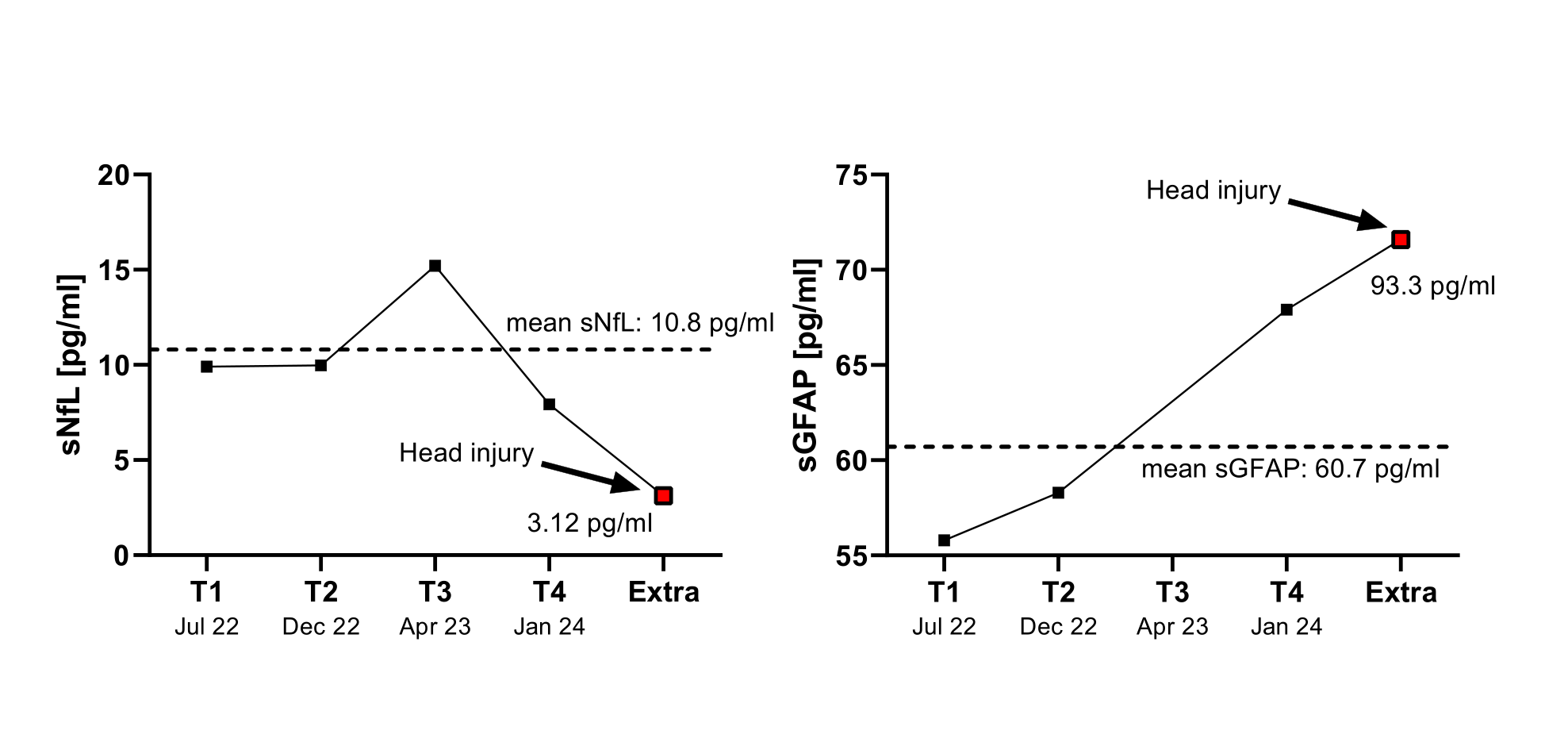

Supplement: Supplementary file 2 — Supplementary file1 (TIFF 122 KB) [file 415_2025_13176_MOESM2_ESM.tiff]
